# Special Issue “Gut Microbioma Structure and Functions in Human Health and Disease”: Editorial

**DOI:** 10.3390/microorganisms11051220

**Published:** 2023-05-06

**Authors:** Francesco Di Pierro

**Affiliations:** Scientific Department, Velleja Research, 20124 Milan, Italy; f.dipierro@vellejaresearch.com

The human gut microbiota is an integral component of the human body that can strike a delicate balance between health and disease [1,2]. Driven by the different aspects of life (delivery, weaning, diet, lifestyle, therapies, environmental triggers, etc.), the gut microbiota is also affected by many host factors (genetics, hormones, pH, bile, pancreatic secretions, etc.). Altogether, these elements contribute to make it temporally malleable and highly personalized [3,4]. How much a peculiar gut microbiota structure is then the cause, or the contributing cause, of disease, or to what extent is simply the consequence of a pathology, remains to be defined. Nevertheless, the possibility that the colonic microbiota can directly contribute to pathological manifestation is being reported increasingly frequently, for example, in inflammatory bowel disease [5]. Undeniably, however, we are at the beginning of a journey that we hope will soon lead us to a better understanding of how the different consortium structures that we observe when analysing the gut microbiota can become useful in a translational sense, providing us with elements on which to work with the aim of improving the health of patients. The Special Issue “Gut Microbioma Structure and Functions in Human Health and Disease” aims to fill in some of the research gaps that still limit us from being able to translate this limited knowledge into everyday medicinal practices.

Since most of the currently available data mainly derive from populations living in a western context, where *Bacteroides* dominate, the research of Di Cristanziano et al. [6] has focused on the in-depth investigation of gut microbiota structure in infants, children, and adults living in sub-Saharan, rural, or semi-urban areas of Côte d’Ivoire, where *Prevotella* dominate in the composition of gut bacteria. Their investigation aimed to delineate a potential link between unicellular parasites (*Blastocystis* and *Entamoeba* spp.), enteric pathogens, and gut microbiota. As previously described [7,8], the authors observed a lower abundance of *Bacteroides* in subjects infected with parasites. This finding could be suggestive of a minor predisposition to host a parasite in *Bacteroides*-driven enterotype subjects [6]; however, the dominance of taxa such as *Prevotella* and *Succinivibrio* could signal the attempt of microbiota to promote a more effective Th2 response [9,10].

As regards to Firmicutes and Bacteroidetes, their geography inside the gut microbiota shows some differences, mostly driven by pH, with Firmicutes being slightly more abundant in the proximal colon and Bacteroidetes slightly more abundant in the distal colon [11,12]. Phipps et al. attempted to assess both the cancer-associated (on) and cancer-adjacent (off) microbiota between right- and left-sided colorectal cancer patients, demonstrating that the presence of a colonic cancer leads to a more consistent microbiota between the two locations [13]. By comparing the bacterial taxa between paired on- and off-cancer microbiota, they showed that patients with right-sided colorectal cancer had a relatively consistent on- and off-cancer microbiota, with only few bacterial taxa being differentially enriched. In contrast, patients with left-sided colorectal cancer had a more varied on- and off-cancer microbiota, showing many differentially enriched bacterial taxa. This could suggest that there is a shift in the left cancer microbiota away from the typical microbiota found in the left colon, becoming more like the right colonic microbiota. This explains why patients with right-sided colorectal cancer develop symptoms later and may also explain why right-sided cancer tends to be more advanced and larger compared to left-sided cancers, as the right colonic microbiota can be primed to support colorectal cancer progression.

Gut microbiota could also have an impact on gastrointestinal infections (GIs), and exclusively breastfed infants appear to be protected against specific infections during the breastfeeding period [14]. Unfortunately, exclusive breastfeeding is not always possible. Pastor-Villaescusa et al. reviewed the role of *L. fermentum* CECT5716 in halting GIs demonstrating that the administration of this strain in milk formulas may significantly prevent enteritis in infants up to 12 months old [15]. This new evidence could in the future extend the list of probiotics suggested by The European Society of Pediatric Gastroenterology, Hepatology, and Nutrition (ESPGHAN) Committee on Nutrition to reduce the risk of GIs in infants [16].

One of the most recognized drivers of the gut microbiota is food. Khine et al. investigated whether foods of different origins in random rotation could destabilise gut microbiota, allowing orally introduced exogenous microbes to be colonised [17]. Their research demonstrated that random rotation in food choice balances the gut microbiota profile, leading to a non-predominant enterotype, and does not alter the capability of gut microbiota to be resistant to new possible colonizers penetrated via an oral route.

As demonstrated by Liu et al., a principal coordinate analysis revealed that the oral microbiota clusters separately from the gut microbiota [18]. At the same time, oral genera are, to some extent, significantly associated with gut genera, and since specific diet are already associated with the presence of specific taxa in the oral microbiota, it is possible that a clear tri-partite relationship between food and both oral and gut microbiota could exist. Liu et al.’s study could provide more precise information than that achieved by simpler studies of the dual relationship between food and gut microbiota.

The gut microbiota has recently emerged as a critical modulator of brain function, and the gut–brain axis is now considered a possible target to better investigate the links existing between neurodegenerative and mental health conditions, including Alzheimer’s disease [19]. Though there is a paucity of human clinical trials, Kang et al. reviewed the state-of-the-art prebiotics that modulate the gut–brain axis, highlighting those that could potentially modify the gut microbiota by promoting the production of butyrate, indoles, and secondary bile acid profiles that further regulate gut immunity and mucosal homeostasis, with beneficial effects on the central immune system and brain functionality [20].

The microbiota is so enormously lively and diverse that it is impossible to consider it to black-and-white terms. In addition, the perspective describing the existence of a “good” and of a “bad” gut microbiota, as well as of beneficial or harmful bacteria, is most likely incorrect and potentially misleading. Furthermore, most publications on microbiota, even if scientifically correct and relevant, show a tendency to overinterpret correlations and sometimes even point towards causality when none is proven. As correctly described by Cani et al. in their review [21], this simplistic and/or overenthusiastic reporting is undoubtedly a major reason why this field is still suffering from major criticism and has failed to convince certain specialists of its validity. As in the classical field of investigation (physiology, biochemistry, etc.), only well-designed experimental procedures and prospective clinical trials will allow researchers to address the many unanswered questions in this field.

## Data Availability

Not applicable.

## References

[1] Falony G., Joossens M., Vieira-Silva S., Wang J., Darzi Y., Faust K., Kurilshikov A., Bonder M.J., Valles-Colomer M., Vandeputte D. (2016). Population-level analysis of gut microbiome variation. Science.

[2] Asnicar F., Berry S.E., Valdes A.M., Nguyen L.H., Piccinno G., Drew D.A., Leeming E., Gibson R., Le Roy C., Khatib H.A. (2021). Microbiome connections with host metabolism and habitual diet from 1,098 deeply phenotyped individuals. Nat. Med..

[3] Stewart C.J., Ajami N.J., O’Brien J.L., Hutchinson D.S., Smith D.P., Wong M.C., Ross M.C., Lloyd R.E., Doddapaneni H., Metcalf G.A. (2018). Temporal development of the gut microbiome in early childhood from the TEDDY study. Nature.

[4] Chen L., Wang D., Garmaeva S., Kurilshikov A., Vich Vila A., Gacesa R., Sinha T., Segal E., Weersma R.K., Lifelines Cohort Study (2021). The long-term genetic stability and individual specificity of the human gut microbiome. Cell.

[5] Ma X., Lu X., Zhang W., Yang L., Wang D., Xu J., Jia Y., Wang X., Xie H., Li S. (2022). Gut microbiota in the early stage of Crohn’s disease has unique characteristics. Gut Pathog..

[6] Di Cristanziano V., Farowski F., Berrilli F., Santoro M., Di Cave D., Glé C., Daeumer M., Thielen A., Wirtz M., Kaiser R. (2021). Analysis of Human Gut Microbiota Composition Associated to the Presence of Commensal and Pathogen Microorganisms in Côte d’Ivoire. Microorganisms.

[7] Andersen L.O., Bonde I., Nielsen H.B., Stensvold C.R. (2015). A retrospective metagenomics approach to studying Blastocystis. FEMS Microbiol. Ecol..

[8] Nieves-Ramírez M.E., Partida-Rodríguez O., Laforest-Lapointe I., Reynolds L.A., Brown E.M., Valdez-Salazar A., Morán-Silva P., Rojas-Velázquez L., Morien E., Parfrey L.W. (2018). Asymptomatic Intestinal Colonization with Protist Blastocystis is Strongly Associated with Distinct Microbiome Ecological Patterns. mSystems.

[9] Castañeda S., Muñoz M., Villamizar X., Hernández P.C., Vásquez L.R., Tito R.Y., Ramírez J.D. (2020). Microbiota characterization in Blastocystis-colonized and Blastocystis-free school-age children from Colombia. Parasites Vectors.

[10] Zhu J., Paul W.E. (2008). CD4 T cells: Fates, functions, and faults. Blood.

[11] Flynn K.J., Ruffin M.T., Turgeon D.K., Schloss P.D. (2018). Spatial Variation of the Native Colon Microbiota in Healthy Adults. Cancer Prev. Res..

[12] Duncan S.H., Louis P., Thomson J.M., Flint H.J. (2009). The role of pH in determining the species composition of the human colonic microbiota. Environ. Microbiol..

[13] Phipps O., Quraishi M.N., Dickson E.A., Steed H., Kumar A., Acheson A.G., Beggs A.D., Brookes M.J., Al-Hassi H.O. (2021). Differences in the On- and Off-Tumor Microbiota between Right- and Left-Sided Colorectal Cancer. Microorganisms.

[14] Duijts L., Jaddoe V.W.V., Hofman A., Moll H.A. (2010). Prolonged and exclusive breastfeeding reduces the risk of infectious diseases in infancy. Pediatrics.

[15] Pastor-Villaescusa B., Blanco-Rojo R., Olivares M. (2021). Evaluation of the Effect of Limosilactobacillus fermentum CECT5716 on Gastrointestinal Infections in Infants: A Systematic Review and Meta-Analysis. Microorganisms.

[16] Wolvers D., Antoine J.M., Myllyluoma E., Schrezenmeir J., Szajewska H., Rijkers G.T. (2010). Guidance for substantiating the evidence for beneficial effects of probiotics: Prevention and management of infections by probiotics. J. Nutr..

[17] Khine W.W.T., Teo A.H.T., Loong L.W.W., Tan J.J.H., Ang C.G.H., Ng W., Lee C.N., Zhu C., Lau Q.C., Lee Y.-K. (2021). Gut Microbiome of a Multiethnic Community Possessed No Predominant Microbiota. Microorganisms.

[18] Liu K., Chen S., Huang J., Ren F., Yang T., Long D., Li H., Huang X. (2021). Oral Microbiota of Children Is Conserved across Han, Tibetan and Hui Groups and Is Correlated with Diet and Gut Microbiota. Microorganisms.

[19] Vogt N.M., Kerby R.L., Dill-McFarland K.A., Harding S.J., Merluzzi A.P., Johnson S.C., Carlsson C.M., Asthana S., Zetterberg H., Blennow K. (2017). Gut Microbiome Alterations in Alzheimer’s Disease. Sci. Rep..

[20] Kang J.W., Zivkovic A.M. (2021). The Potential Utility of Prebiotics to Modulate Alzheimer’s Disease: A Review of the Evidence. Microorganisms.

[21] Cani P.D., Moens de Hase E., Van Hul M. (2021). Gut Microbiota and Host Metabolism: From Proof of Concept to Therapeutic Intervention. Microorganisms.

